# Immune and Epstein-Barr virus gene expression in cerebrospinal fluid and peripheral blood mononuclear cells from patients with relapsing-remitting multiple sclerosis

**DOI:** 10.1186/s12974-015-0353-1

**Published:** 2015-07-14

**Authors:** Caterina Veroni, Fabiana Marnetto, Letizia Granieri, Antonio Bertolotto, Clara Ballerini, Anna Maria Repice, Lucia Schirru, Giancarlo Coghe, Eleonora Cocco, Eleni Anastasiadou, Maria Puopolo, Francesca Aloisi

**Affiliations:** Department of Cell Biology and Neuroscience, Istituto Superiore di Sanità, Viale Regina Elena 299, 00161 Rome, Italy; Neurology 2—CRESM (Multiple Sclerosis Regional Reference Center), AOU San Luigi Gonzaga, Regione Gonzole 10, 10043 Orbassano, Italy; Department of Neuroscience, Drug and Child Health (NEUROFARBA), University of Florence, Viale Pieraccini 6, 50137 Florence, Italy; Multiple Sclerosis Center, Neurology 2 Division, Careggi University Hospital, University of Florence, Viale Morgagni 85, 50134 Florence, Italy; Department of Public Health, Clinical and Molecular Medicine, Multiple Sclerosis Center, University of Cagliari, Via Is Guadazzonis, 2, 09126 Cagliari, Italy; Department of Experimental Medicine, La Sapienza University, Viale Regina Elena 324, 00161 Rome, Italy; Department of Pathology, Beth Israel Deaconess Medical Center/Harvard Medical School, 330 Brookline Avenue, Boston, MA 02215 USA

**Keywords:** Multiple sclerosis, Immune response, Epstein-Barr virus, Gene expression, Biomarkers, Multivariate analysis

## Abstract

**Background:**

Gene expression analyses in paired cerebrospinal fluid (CSF) and peripheral blood mononuclear cells (PBMC) from patients with multiple sclerosis (MS) are restrained by the low RNA amounts from CSF cells and low expression levels of certain genes. Here, we applied a Taqman-based pre-amplification real-time reverse-transcription polymerase chain reaction (RT-PCR) (PreAmp RT-PCR) to cDNA from CSF cells and PBMC of MS patients and analyzed multiple genes related to immune system function and genes expressed by Epstein-Barr virus (EBV), a herpesvirus showing strong association with MS. Using this enhanced RT-PCR method, we aimed at the following: (1) identifying gene signatures potentially useful for patient stratification, (2) understanding whether EBV infection is perturbed in CSF and/or blood, and (3) finding a link between immune and EBV infection status.

**Methods:**

Thirty-one therapy-free patients with relapsing-remitting MS were included in the study. Paired CSF cells and PBMC were collected and expression of 41 immune-related cellular genes and 7 EBV genes associated with latent or lytic viral infection were determined by PreAmp RT-PCR. Clinical, radiological, CSF, and gene expression data were analyzed using univariate and multivariate (cluster analysis, factor analysis) statistical approaches.

**Results:**

Several immune-related genes were differentially expressed between CSF cells and PBMC from the whole MS cohort. By univariate analysis, no or only minor differences in gene expression were found associated with sex, clinical, or radiological condition. Cluster analysis on CSF gene expression data grouped patients into three clusters; clusters 1 and 2 differed by expression of genes that are related mainly to innate immunity, irrespective of sex and disease characteristics. By factor analysis, two factors grouping genes involved in antiviral immunity and immune regulation, respectively, accurately discriminated cluster 1 and cluster 2 patients. Despite the use of an enhanced RT-PCR method, EBV transcripts were detected in a minority of patients (5 of 31), with evidence of viral latency activation in CSF cells or PBMC and of lytic infection in one patient with active disease only.

**Conclusions:**

Analysis of multiple cellular and EBV genes in paired CSF cell and PBMC samples using PreAmp RT-PCR may yield new information on the complex interplay between biological processes underlying MS and help in biomarker identification.

**Electronic supplementary material:**

The online version of this article (doi:10.1186/s12974-015-0353-1) contains supplementary material, which is available to authorized users.

## Background

Multiple sclerosis (MS) is the most common chronic inflammatory disease of the central nervous system (CNS) leading to demyelination, axonal damage, and neuronal loss [1, 2]. The diagnosis of MS relies on clinical symptoms, magnetic resonance imaging (MRI) findings, and laboratory tests, such as detection of oligoclonal bands in the cerebrospinal fluid (CSF) [3]. MS has a heterogeneous and unpredictable clinical course spanning decades; the different rates of progression and the different responses of patients with relapsing-remitting MS (RRMS) to therapy remain unexplained.

It is widely accepted that MS pathology is caused by an inappropriate T-cell-mediated immune response that is induced in secondary lymphoid organs upon encounter with still unknown antigens [4]. Leukocyte migration and activation inside the brain and spinal cord is accompanied by persistent intrathecal B-cell activation and antibody production whose role in MS pathology is not understood yet [5]. At the cellular level, MS-associated inflammation is characterized by mild-to-moderate CSF pleiocytosis, perivascular accumulation of leukocytes (predominantly lymphocytes and myeloid cells) in the white matter and in the meninges, organization of lymphoid-like structures in the subarachnoid space, and microglia/macrophage activation in the neural parenchyma [1, 2]. The relationship between peripheral immune system activation and CNS inflammation is highlighted by the therapeutic efficacy of natalizumab, which blocks leukocyte trafficking in the CNS and markedly reduces disease activity and leukocyte number, cytokine, and IgG levels in CSF [6–9].

The identification of sensitive and specific biomarkers for diagnosis, prognosis, and treatment efficacy of MS is a relentless effort [10]. At the protein level, CSF biomarkers for inflammation, like the B-cell attracting chemokine C-X-C motif chemokine 13 (CXCL13) and the extracellular matrix-degrading enzyme metalloprotease-9 (MMP-9) [11], and for CNS tissue damage, like myelin basic protein and neurofilament light chain subunit [12], have been identified. It has been shown that CSF levels of chitinase-3-like-1 [13, 14] and neurofilament light [13] chain are significant predictors of MS development and neurological disability. Several microarray-based gene expression studies have been carried out in whole blood or peripheral blood mononuclear cells (PBMC), aiming at detecting differences in gene signatures between MS patients and control subjects and between patients with clinically or radiologically active and inactive disease [15–20]. However, owing to small sample size, disease heterogeneity, and differences in microarray technology and data analysis, reproducibility across studies has been extremely limited [21]. Due to the small number of cells collected from the CSF, comprehensive gene expression studies in CSF cells are sparse. Compared with healthy controls or patients with non-inflammatory neurological diseases, MS patients show increased expression of genes involved in T-, NK-, and B-cell function in CSF cells [17, 22–24]. Only a few studies have examined gene expression in paired CSF cells and PBMC from patients with MS and non-inflammatory neurological diseases, confirming poor correlation between intrathecal and peripheral immune activation [17, 22, 23].

Real-time reverse-transcription polymerase chain reaction (RT-PCR) incorporating a target gene pre-amplification (PreAmp) step has the double advantage to improve detection of low-frequency transcripts and to enable analysis of a large number of transcripts even with low amounts of starting RNA [25]. Here, we report the application of this enhanced RT-PCR method to paired CSF cell and PBMC samples from patients with RRMS and the results of a preliminary analysis on nearly 50 genes, including immune-related genes and genes expressed by Epstein-Barr virus (EBV). This ubiquitous DNA herpesvirus establishes a life-long latent infection and shows strong association with MS [26, 27]. MS risk is higher after infectious mononucleosis, and immune reactivity to EBV is increased or deregulated in MS patients compared to healthy subjects, indicating a disturbance in virus-host interactions [27–29]. It is debated whether an active EBV infection in the CNS of MS patients can cause an immunopathological response [27, 30–36]. Although EBV DNA load in CSF and peripheral blood does not differ significantly between MS patients and healthy donors or patients with other neurological diseases [37–40], some studies support an association between increased EBV load in peripheral blood and clinical MS attacks [38, 41, 42]. It has not been established yet whether the study of EBV gene expression, which defines more precisely the different phases of viral infection [43], might be a better strategy to investigate EBV perturbation in MS [38, 44].

Thus, the goal of this study has been to evaluate whether the combined analysis of immune-related and EBV genes in CSF cells and PBMC obtained from clinically and radiologically characterized, therapy-free RRMS patients could provide novel information on the relationship between immune status, EBV infection, and MS disease features. After analysis of the expression levels of each selected gene according to sex, clinical, MRI, and CSF findings, all the collected data have been extensively analyzed using multivariate statistical methods in the attempt to identify gene expression patterns representative of underlying immunopathological processes and potentially useful for patient classification.

## Methods

### Subjects

Patients were recruited at the MS centers of the University Hospital San Luigi Gonzaga in Orbassano, University of Cagliari, and University of Firenze. The study was approved by the ethic committees of the three participating MS centers and of the Istituto Superiore di Sanità, and carried out according to the Declaration of Helsinki. Written informed consent was obtained from all study participants.

Thirty-one patients with RRMS were included in this study [45, 46]. None of the patients received immunomodulatory or immunosuppressive treatment at the time of CSF and blood sample collection and had not received such treatments for at least 12 months. Demographic and clinical information were derived from medical records and are summarized in Table 1. MS disease onset was defined as the first episode of focal neurological dysfunction indicative of MS; relapses were defined as the development of new or recurrent neurological symptoms not associated with fever or infection and lasting for at least 24 h [46]. Patients were categorized on the basis of the presence of a relapse or a condition of remission at the time of sampling. The expanded disability status scale (EDSS) score was calculated on the basis of a complete neurological examination by a neurologist expert in MS.

**Table 1:** Demographic, clinical, and CSF data of the analyzed patients

	Female/male (ratio)	Age, median (range)	EDSS, median (range)	Time since diagnosis, median (range)	Patients in clinical relapse, *n* (%)	Patients with Gd+ lesions on MRI, *n* (%)	Number of cells/μl CSF, median (range)	IgG index, median (range)
Relapsing remitting MS (*n* = 31)	20/11 (1.8)	33 years (20–65)	1 (0–4.5)	12 months (0.1–144)	12 (38.7)	13 (41.9)	7 (0.5–45)	0.81 (0.43–2.2)

All patients were examined by a routine brain MRI protocol before or after sample collection (median time interval =26 days; range 1–90 days). The time interval between sample collection and MRI was significantly shorter in patients in clinical relapse (median =11 days, range 1–37 days) than in patients in clinical remission (median =42 days, range 2–90) (*p* = 0.0015 by Student’s t test). MRI scans (T2-weighted and T1-weighted pre- and post-gadolinium administration, slice thickness 5 or 3 mm) were obtained in all patients using a standardized scanning protocol [47] with 1.5 T MR scanners.

### Sample collection

All CSF and peripheral blood samples were obtained for routine diagnostic work-up. CSF and blood from each patient were always drawn on the same day. CSF samples were processed according to the BioMS-eu consortium guidelines [48]. A total of 3 to 17 ml of CSF (median =11 ml) were obtained by lumbar spinal tap with an atraumatic needle. Within 30 min after lumbar puncture, CSF samples were centrifuged at 1200 rpm for 10 min at room temperature to separate the cellular component from cell-free supernatant; the cell pellets were stored at −80 °C in RNAlater (Qiagen) or RNA was immediately extracted (see below). Blood (10 ml) was drawn in EDTA tubes, and PBMC were isolated using Lymphoprep, preserved in RNAlater and frozen at −80 °C. CSF samples were routinely analyzed for cell counts. Quantitative (IgG index) and qualitative (oligoclonal bands) analysis of intrathecal IgG synthesis after lumbar puncture was performed using standard methods.

### Pre-amplification real-time RT-PCR

Total RNA was extracted from CSF cells (median =4.5 × 10^4^, range 7 × 10^3^–5 × 10^5^) and PBMC (4 × 10^5^) using the AMBION RNAqueous micro kit (Life Technologies, Grand Island, NY, USA) according to the manufacturer’s instructions, including genomic DNA digestion. Total RNA from PBMC was quantified by Nanodrop 2000 (Thermo Fisher Scientific, Waltham, MA, USA) and 200 ng was reverse transcribed for each sample. Because of the very low and highly variable RNA yield from CSF cells, the entire volume (15 μl) of RNA extracted from each CSF sample was reverse transcribed. Reverse-transcription (RT) was performed using the high capacity reverse transcription kit with RNase inhibitor (Life Technologies). The resulting cDNA was diluted to a final volume of 50 μl and splitted into four 12.5 μl aliquots. To increase the number of targeted copies, each cDNA aliquot was amplified for the specific gene assays by pre-amplification reaction (14 cycles) using the TaqMan PreAmp Master Mix (Life Technologies) and pooled gene-specific primers, and the reaction conditions indicated by the manufacturer. Inventoried and self-designed TaqMan gene expression assays were used to study cellular and EBV genes, respectively (see Additional files 1 and 2). Cellular gene assays were pre-amplified together with the housekeeping gene GAPDH; viral gene assays were pre-amplified separately together with GAPDH and the B-cell-specific genes CD19 and CD20. The pre-amplification product was diluted 1:5 up to 250 μl in TE buffer, and 4 μl of this dilution was used as template for a single real-time PCR analysis. Quantitative PCR experiments were performed in triplicates with the same inventoried or self-designed TaqMan assays used in the pre-amplification step (250 nM probe and 900 nM each primer), using the 7500 Real-Time PCR System (Life Technologies) for cellular genes and the StepOne Plus Real-Time PCR System (Life Technologies) for viral genes. Thermocycling parameters were 50 °C (2 min), 95 °C (10 min), followed by 40 cycles of 95 °C (15 s) and 60 °C (1 min) for both cellular and viral genes. The results of gene expression analysis are expressed as Ct values (Ct = threshold cycle of PCR at which the amplified product is detected). The ΔCt is the difference in Ct values derived from the gene of interest and the reference gene GAPDH; the factor 2^-ΔCt is used to express the ratio between the gene of interest and the internal reference gene. To rule out cross-contamination of reagents and primers, all RT, pre-amplification, and real-time PCR experiments included a NTC sample, containing all the components of each reaction except for the template. Considering that 12 μl of pre-amplified cDNA was analyzed for each transcript and that the available volume of each pre-amplified aliquot was 250 μl, we were able to analyze in triplicates up to 20 transcripts per aliquot.

To check that all amplicons were amplified uniformly without bias, we performed pre-amplification uniformity experiments using non-limiting cDNA from a human non pathological pulmonary hilar lymph node (obtained from Dr. Egidio Stigliano, Institute of Pathological Anatomy, Policlinico A. Gemelli, Rome, Italy), as control for cellular genes, and from an EBV transformed B-lymphoblastoid cell line (LCL), as control for EBV genes. The EBV+ LCL (L5) was generated by infecting 5 × 10^6^ PBMC obtained from a patient with MS with B95.8 EBV strain in a medium containing cyclosporin A (1 μg/ml, Calbiochem); the outgrowth of B95.8-infected PBMC was monitored twice a week, and after 5 weeks post-infection, the LCL was permanently established. Amplification of pre-amplified cDNA from lymph node and EBV+ LCL was compared with that of non pre-amplified cDNA. Primer uniformity was calculated by the formula ΔΔCt = ΔCt (Preamp) − ΔCt (cDNA). A ΔΔCt value within ±1.5 is considered acceptable, as indicated in the manufacturer’s instructions. Pre-amplification uniformity values related to the reference gene GAPDH were very close to zero for all the investigated gene assays (mean ΔΔCt values ± SD were 0.33 ± 0.35 and 0.90 ± 0.41 for EBV and cellular transcripts, respectively), indicating optimal pre-amplification uniformity. PCR efficiency by direct and PreAmp real-time PCR was checked for viral genes and found to be similar (range 0.97–1.08; optimal efficiency = 100 ± 10 %) over serial dilutions of EBV+ LCL cDNA (from 100 to 0.1 ng, corresponding to approximately 10.000 to 1 cells). Importantly, similar data were obtained for each target gene after pre-amplification from pooled and single assays.

### Statistical analysis

Demographic, clinical, radiological, CSF, and gene expression data of 31 MS patients were analyzed by univariate and multivariate statistical techniques. In univariate analyses, comparisons between groups of patients were carried out by Student’s t test and Mann-Whitney test for continuous variables, and by Fisher’s exact probability test for categorical variables. Correlation between variables was assessed by Spearman’s rank correlation coefficient. Means and SE, or medians and interquartile ranges, were used to summarize continuous data, and percentages were used for categorical variables.

To unravel complex gene interactions that may better capture pathological processes in MS, the collected gene expression data were analyzed using two multivariate statistical techniques: cluster analysis aiming to group subjects into clusters and factor analysis aiming to define artificial factors, that is underlying latent variables, adequately describing the correlation structure of the original variables. Cluster analysis was carried out by average linkage method with Euclidean similarity measure. Clustering of patients was visualized by dendrogram and the choice of number of groups was based on Calinski/Harabasz pseudo-F index and Duda/Hart index stopping rules. Factor analysis was carried out using the principal factor method. Factor loadings, that is, correlations of the original variables with factors, were used for interpretation of artificial factors. Scores of subjects on artificial factors were entered in further analyses. The influence of demographic, clinical, or radiological parameters on patient clustering and factor scores was investigated by univariate analyses. Finally, the discriminating power of artificial factors to predict patient clustering was assessed. Classification accuracy was assessed through receiver operating characteristic (ROC) curve analysis, by calculating the area under ROC curves (AUC) and its 95 % confidence interval (CI). These analyses were carried out separately on immune gene expression data obtained in CSF and PBMC samples.

The level of confidence was set at 0.05 and statistical significance was assessed by adopting the Bonferroni correction for multiple testing. Number of comparisons considered patient subgroups differing for demographic, clinical, and radiological characteristics, and resulting from cluster analysis. For correlation analyses, multiplicity due to two inflammatory CSF parameters (IgG index and CSF cell count) was considered. Stata 11 was used for statistical analyses.

## Results

### Setup of PreAmp real-time RT-PCR

**Fig. 1:**
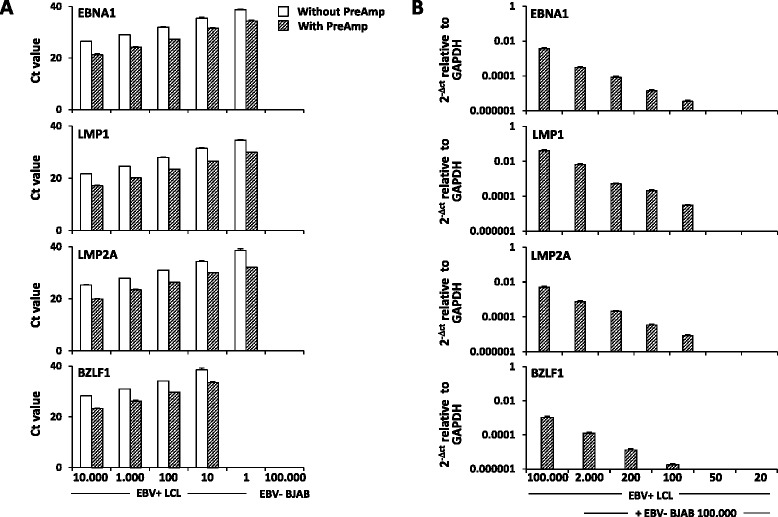
Improved sensitivity and specificity of PreAmp real-time RT-PCR for EBV transcript detection. **a** The expression levels of three EBV latent genes (EBNA1, LMP1, LMP2A) and one EBV lytic gene (BZLF1) were investigated in serially diluted EBV transformed (EBV+) LCL and in the EBV negative (EBV−) B lymphoma cell line BJAB, with and without pre-amplification (PreAmp), using Taqman self-designed gene assays. PreAmp resulted in an improvement of 4.1 to 5.1 cycles within the threshold Ct for low gene expression levels (≤35). EBV latent and lytic transcripts were detectable down to 1 and 10 EBV+ LCL cells, respectively; no signal was detected in EBV− BJAB cells confirming assay specificity. **b** Pre-Amp RT-PCR was applied to cDNA from EBV+ LCL cells that were serially diluted in a background of EBV− BJAB cells; the lower limits of detection of latent and lytic transcripts were one and two EBV+ LCL cells in 1 × 10^4^ EBV− negative cells, respectively

In preliminary experiments, the TaqMan® PreAmp Master Mix technique was applied to cDNA obtained from human lymphoid tissue and an EBV+ lymphoblastoid cell line (L5), as positive controls for immune-related and viral genes, respectively. Robust pre-amplification of multiple-pooled Taqman ABI inventoried gene assays (41 cellular genes listed in Additional file 1) and self-designed gene assays (7 EBV genes listed in Additional file 2) was obtained with a mean improvement of 4.6 ± 0.4 cycles (range 4.1–5.1) (*p* < 0.0001 compared to the Ct values obtained with the direct real-time RT-PCR). Figure 1 shows the specificity and increased sensitivity of PreAmp RT-PCR and the lower limits of detection of four of the seven EBV gene expression assays tested in the EBV+ LCL.

### Gene expression in CSF cells and PBMC from RRMS patients

PreAmp real-time RT-PCR was then applied to cDNA from CSF cells and PBMC collected from 31 therapy-free RRMS patients. Thirty-one CSF and 29 PBMC samples were eligible for RNA analysis. The demographic, clinical, and CSF characteristics of the MS cohort are presented in Table 1. Twelve patients (38.7 %) experienced a clinical relapse at the time of CSF and blood sampling, and 13 patients (41.9 %) had gadolinium enhancing lesions on the brain MRI.

### Immune-related gene expression

The selected set of immune-related genes includes the following: T-lymphocyte (CD4, CD8, forkhead box P3 (FoxP3)), B-lymphocyte (CD19, CD20, CD138, and B-cell maturation antigen (BCMA), the receptor for B-cell activating factor (BAFF) and a proliferation-inducing ligand expressed on B-lineage cells), natural killer (NK) cell (CD56, NK cell p46-related protein (NKp46)), monocyte/macrophage (CD68) and plasmacytoid dendritic cell (pDC) (blood dendritic cell antigen 2 (BDCA-2)) markers; granzyme B and perforin, the lytic enzymes mediating T cell/NK cell cytotoxic activity; cytokines of T-cell and/or NK-cell origin (interferon-γ (IFN-γ), interleukin (IL)-2, IL-4, IL-5, IL-17A) and of predominantly macrophage/DC origin (tumor necrosis factor (TNF), IL-1β, IL-6, IL-10, p40 (cytokine subunit shared by IL-12 and IL-23), IL-15); major histocompatibility complex (MHC) class II, involved in antigen presentation; the enzyme MMP-9 involved in extracellular matrix and myelin degradation; the enzyme nicotinamide phosphoribosyltransferase (NAMPT) involved in inflammation, metabolic, and stress responses; COX-2, the enzyme responsible for prostaglandin synthesis at sites of inflammation; inducible nitric oxide synthase (iNOS), the enzyme involved in the generation of nitric oxide; the BAFF; and C-X-C motif chemokine 10 (CXCL10), an IFN-inducible chemoattractant for activated T cells and NK cells, and CXCL13, a B-cell chemoattractant; molecules involved in type-1 IFN production (interferon regulatory factor 7 (IRF7)) and binding (IFN-α-inducible protein 6 (IFN-αR1)) and induced by type-1 IFN (interferon-stimulated exonuclease gene 20 kDa (ISG20), myxovirus (influenza virus) resistance protein (MxA), 2′-5′-oligoadenylate synthetase 1 (OAS1), protein kinase R (PKR), IFN-induced protein with tetratricopeptide repeats (IFIT1), IFN-α-inducible protein 6 (IFI6), ubiquitin-specific peptidase 18 (Usp18)). Most of the investigated immune transcripts were detected in all or the majority (>80 %) of CSF and PBMC samples (Table 2). A few transcripts, like IL-17A, IL-4, p40, CXCL13, and iNOS, were detected in a lower percentage of CSF cell and/or PBMC samples; only IL-5 was always undetectable (Table 2).

**Table 2:** Expression of immune-related genes in CSF cells and PBMC from RRMS patients

Gene	CSF cell samples with detectable gene expression (%)	Relative amount in CSF cells, median (range)^a^	PBMC samples with detectable gene expression (%)	Relative amount in PBMC, median (range)^a^	Differences in gene expression between CSF cells and PBMC^b^
CD20	100	0.28 (0.05–2.23)	100	0.39 (0.05–9.87)	n.s.
CD19	97	0.052 (0–0.18)	100	0.041 (0.007–0.18)	n.s.
CD138	94	0.033 (0–0.75)	79	0.00014 (0–0.0016)	<0.0001
BCMA	97	0.017 (0–0.15)	97	0.0019 (0–0.019)	<0.0001
CD4	100	0.74 (0.30–2.44)	100	0.45 (0.014–3.04)	0.0005
CD8	100	1.22 (0.31–4.93)	100	0.86 (0.04–2.27)	0.012
CD56	94	0.015 (0–0.026)	97	0.012 (0–0.90)	n.s.
NKp46	90	0.018 (0–0.083)	100	0.025 (0.002–0.129)	n.s.
CD68	100	0.29 (0.007–2.02)	100	0.77 (0.04–6.8)	0.0002
FoxP3	97	0.21 (0–2.42)	100	0.059 (0.00002–0.27)	0.0003
BDCA-2	97	0.057 (0–0.42)	100	0.019 (0.001–0.24)	0.0002
Perforin	100	0.18 (0.03–0.70)	100	0.21 (0.029–7.35)	n.s.
Granzyme B	97	0.014 (0–0.058)	100	0.068 (0.003–2.51)	<0.0001
MMP-9	84	0.006 (0–0.27)	100	0.19 (0.014–9.87)	<0.0001
IFN-γ	94	0.01 (0–0.079)	97	0.004 (0–0.089)	0.0064
TNF	100	0.10 (0.03–1.56)	100	0.064 (0.006–2.1)	0.02
IL-1β	97	0.089 (0–6.4)	100	0.45 (0.019–27.8)	0.02
IL- 2	84	0.0016 (0–0.015)	90	0.0007 (0–0.005)	0.03
IL-4	48	0 (0–0.0004)	69	0.00004 (0–0.0009)	0.004
IL-5	0	0	0	0	–
IL-6	84	0.0017 (0–0.14)	97	0.008 (0–0.16)	0.0015
IL-10	97	0.016 (0–0.22)	100	0.005 (0.0001–0.049)	0.0076
IL-15	87	0.026 (0–0.093)	97	0.02 (0–0.12)	n.s.
IL-17A	23	0 (0–0.0003)	41	0 (0–0.004)	n.s.
p40	77	0.0006 (0–0.046)	59	0.00001 (0–0.0015)	0.003
CXCL10	81	0.0008 (0–0.033)	93	0.0017 (0–0.43)	n.s.
CXCL13	81	0.002 (0–0.043)	66	0.00002 (0–0.0005)	<0.0001
IRF7	100	0.10 (0.027–0.64)	100	0.022 (0.001–1.65)	0.0006
ISG20	100	1.46 (0.17–6.32)	100	0.59 (0.06–7.0)	0.018
IFI6	100	0.18 (0.023–1.96)	100	0.10 (0.01–10.0)	n.s.
MxA	100	0.67 (0.11–6.20)	100	0.24 (0.046–14.39)	0.046
PKR	100	0.36 (0.058–1.91)	100	0.12 (0.006–1.39)	0.023
OAS1	100	0.05 (0.027–0.44)	100	0.038 (0.018–0.90)	n.s.
IFIT1	81	0.0006 (0–0.019)	90	0.001 (0–0.18)	0.0089
Usp18	94	0.008 (0–0.052)	93	0.003 (0–0.26)	0.0025
IFN-αR1	97	0.017 (0–0.053)	97	0.010 (0–0.18)	n.s.
BAFF	100	0.041 (0.010–0.21)	100	0.051 (0.014–1.03)	0.01
NAMPT	100	0.086 (0.016–0.72)	100	0.37 (0.02–19.9)	0.001
MHC class II	100	3.40 (0.66–9.13)	100	3.0 (0.18–92.1)	n.s.
iNOS	52	0.000007 (0–0.0015)	59	0.000005 (0–0.0004)	n.s.
COX-2	94	0.046 (0–0.26)	100	0.22 (0.007–2.15)	0.0008

*n.s.* not significant

^a^Gene expression values are presented as 2^-ΔCt relative to GAPDH. Data obtained in 31 CSF cell and 29 PBMC samples from 31 RRMS patients are shown

^b^Comparisons between paired CSF cell and PBMC samples (available for 29 patients) were made by Wilcoxon signed-rank test

#### Differences in immune-related gene expression between CSF cells and PBMC and correlation with inflammatory CSF parameters

Comparison of gene expression values in paired CSF and PBMC samples available from 29 RRMS patients revealed significantly higher signals for CD138 and BCMA (*p* < 0.0001) in CSF cells and of CD68 (*p* = 0.0002) in PBMC (Table 2), mirroring the well-documented enrichment in plasmablasts and the paucity of monocytes/macrophages in CSF compared to peripheral blood [49]. Other transcripts that were enriched in CSF cells compared to PBMC with very high statistical significance were CD4, FoxP3, BDCA-2, IFN-γ, IL-10, p40, CXCL13, IRF7, and Usp18 (Table 2), in part confirming knowledge of intrathecal immune cell recruitment and cytokine production acquired in flow cytometry and ELISA studies [50–55]. Statistically, highly significant enrichment of granzyme B, MMP-9, IL-4, IL-6, IFIT1, NAMPT, and COX-2 transcripts was found in PBMC compared to CSF cells (Table 2).

Using Spearman’s rank correlation analysis, significant correlations were found between inflammatory CSF parameters and gene expression levels in CSF cells, but not PBMC. CSF cell number correlated positively with the B-cell maturation markers BCMA (*r* = 0.49, *p* = 0.005) and CD138 (*r* = 0.44, *p* = 0.013) and negatively with the macrophage marker CD68 (*r* = −0.65, *p* = 0.0001) and the anti-inflammatory cytokine IL-10 (*r* = −0.52, *p* = 0.003). IgG index correlated positively with CD20 (*r* = 0.49, *p* = 0.005) and negatively with IL-2 (*r* = −0.43, *p* = 0.016). These data are in line with the results of flow cytometry studies in MS patients showing that the B-cell/monocyte ratio is the most variable cell parameter in CSF [49, 55] and that accumulation of B cells/plasmablasts in the CSF correlates with inflammatory CSF parameters [49–51].

#### Differences in immune-related gene expression among MS patients

**Fig. 2:**
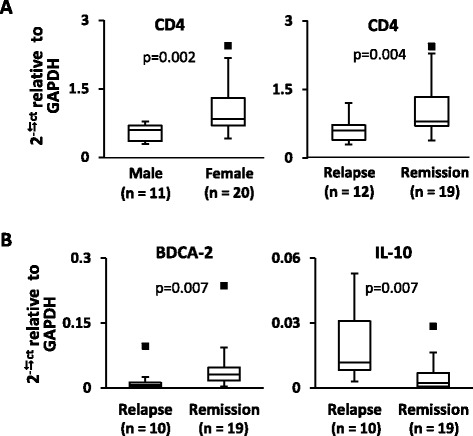
Immune-related genes differentially expressed in CSF cells and PBMC from RRMS patients grouped according to sex and clinical status. Gene expression levels were measured in CSF cells **(a)** and PBMC **(b)** from 31 and 29 RRMS patients, respectively, using PreAmp RT-PCR. The values obtained were compared between patient groups differing for sex (female/male), clinical (relapse/remission), and MRI status (presence/absence of gadolinium-enhancing lesions). Differences between groups were evaluated by Mann-Whitney test; only statistically significant differences (*p* < 0.0125 to account for multiple comparisons) are shown. The *lines inside the boxes* represent the median value; *boxes* extend from the 25th to the 75th percentile, covering the interquartile range (IQR), and *whiskers* extend from 25th percentile −1.5 IQR to the 75th percentile +1.5 IQR. Maximum outliers outside the whiskers are represented by individual marks

Data analysis was then carried out using two different approaches, namely: (1) differences in gene expression levels between RRMS patients differing for demographic, clinical, and radiological features from an univariate point of view; and (2) in a multivariate approach using cluster and factor analyses. Overall, univariate analysis revealed no or only a few differentially expressed genes in both CSF cells and PBMC. Females and clinically remitting MS patients displayed significantly higher expression of CD4 in CSF cells compared to males and clinically relapsing patients, respectively (Fig. 2a). In PBMC, higher expression of BDCA-2 and IL-10 genes was associated with clinical remission and relapse, respectively (Fig. 2b).

**Fig. 3:**
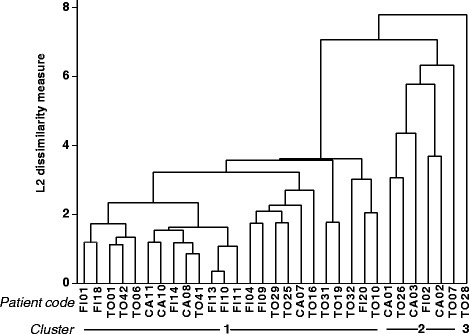
Dendrogram of RRMS patients based on immune gene expression in CSF cells. Cluster analysis was carried out on the expression data of 41 immune-related genes obtained in 31 CSF cell samples, by using average linkage method with Euclidean similarity measure

**Fig. 4:**
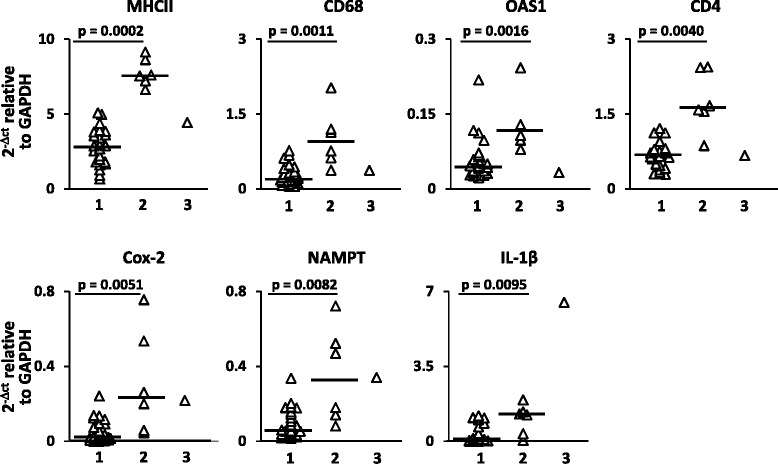
Genes with discriminatory power in cluster analysis. Gene expression values for MHC class II, CD4, CD68, OAS-1, COX-2, NAMPT, and IL-1β in CSF cell samples from RRMS patients clustering into groups 1, 2, and 3 are shown. Significant differences in gene expression between group 1 (*n* = 24) and group 2 (*n* = 6) patients were assessed by Mann-Whitney test; *p* values ≤0.0125 are shown. Each *dot* represents the gene expression value obtained in each individual patient; the *line* marks the median value

Cluster analysis on CSF gene expression data divided RRMS patients into three clusters including 24, 6, and 1 subject, respectively (dendrogram shown in Fig. 3). Compared to cluster 1 (*n* = 24), cluster 2 (*n* = 6) displayed significantly higher signals for MHC class II, CD68, the type-1 IFN-induced gene OAS1, CD4 and indicators of inflammation and macrophage activation like COX-2, NAMPT, and IL-1β (Fig. 4). ROC curve analyses highlighted that, among differentially expressed genes, MHC class II, CD4, CD68, and OAS1 genes showed the best discriminatory accuracy for cluster 1 and cluster 2 (AUC >0.90 by ROC curve analysis) (Table 3). Conversely, cluster 1 and cluster 2 did not differ significantly for demographic (age, sex), clinical (relapse/remission, EDSS), radiological (absence/presence of gadolinium enhancing lesions), or CSF (cell number, IgG index) characteristics. It should however be noted that cluster 2 comprises only female patients in clinical remission. It is also worth mentioning that the only patient in cluster 3 (a male in clinical remission and with inactive MRI) displayed the highest levels of CSF transcripts for TNF, IL-1β, IL-6, IL-17A, MMP-9, and CXCL10 within the study population (see Fig. 4 for IL-1β gene expression values).

**Table 3:** Discriminatory power for patient clustering of genes expressed in CSF cells

Genes	AUC (95 % CI)
MHC class II	1.0 (1.0–1.0)
CD4	0.97 (0.91–1.0)
CD68	0.94 (0.84–1.0)
OAS1	0.92 (0.83–1.0)
COX-2	0.87 (0.72–1.0)
NAMPT	0.85 (0.68–1.0)
IL-1β	0.85 (0.63–1.0)

ROC curve analysis was performed to define the accuracy of genes differentially expressed in CSF cells to discriminate between cluster 1 and cluster 2 patients; cluster 3 patient was not considered

*AUC* area under ROC curve, *CI* confidence interval

Factor analysis on CSF gene expression values identified four artificial factors that explained 26, 16, 13, and 10 % of the variability in the dataset, respectively. Table 4 displays the genes with the strongest correlation with each factor. Factor 1 strongly correlated (factor loadings ≥0.60) with most of the analyzed type-1 IFN-related genes (the transcription factor IRF7 which is activated upon viral nucleic acid binding to Toll-like receptor (TLR)-7 and TLR-9 and regulates type-1 IFN production; the type-1 IFN-stimulated genes MxA, PKR, Usp18, OAS1, IFI6, and IFIT1, and the type-1 IFN receptor subunit IFN-αR1), the IFN-induced B-cell growth factor BAFF, IFN-γ, the cytotoxic T-cell marker CD8 and the inflammatory markers NAMPT, and COX-2, indicating a strong contribution of innate and adaptive antiviral immunity to this factor. Although at a lower level (factor loadings ranging from 0.50 to 0.60), factor 1 was also associated with BDCA-2, perforin, CD4, FoxP3, MHC class II, IL-1β, and TNF. Factor 2 strongly correlated with myeloid cell/macrophage products (IL-1β, IL-6, CXCL10, TNF, MMP-9) and IL-17A (factor loadings ≥0.70), and to a lesser extent (factor loadings ranging from −0.60 to 0.52), with IL-15, perforin, and NKp46, the latter two genes showing an inverse association. Factor 3 was mainly associated with CD20, CD19, ISG20, FoxP3, and p40 (factor loadings >0.60), and to a lesser extent with BCMA, CD8, and IFIT1. Factor 4 was mainly associated with CD68, IL-10, and MHC class II (factor loadings >0.60), and to a lesser extent, with CD4 (factor loading =0.56), suggesting that this factor may describe an immune regulatory response involving IL-10-producing macrophages and T helper cells. None of the analyzed genes contributed to more than one factor with a factor loading >0.60.

**Table 4:** Factor loadings on CSF gene expression data

Gene	Factor 1	Factor 2	Factor 3	Factor 4
MxA	0.90			
IRF7	0.82			
PKR	0.80			
Usp18	0.80			
OAS1	0.75			
NAMPT	0.74			
IFI6	0.73			
BAFF	0.68			
IFN-αR1	0.66			
IFN-γ	0.61			
CD8	0.60		0.51	
IFIT1	0.60		−0.51	
COX-2	0.60			
BDCA-2	0.59			
IL-1β	0.50	0.79		
IL-6		0.79		
CXCL10		0.78		
TNF	0.52	0.72		
IL-17A		0.70		
MMP-9		0.70		
CD20			0.78	
ISG20			0.76	
CD19			0.74	
FoxP3	0.52		0.73	
p40			0.67	
CD68				0.75
IL-10				0.70
MHC class II	0.54			0.68
Perforin	0.58	−0.60		
CD4	0.56			0.56
BCMA			0.54	
IL-15		0.52		
NKp46		−0.50		

Factor loadings >0.5 are shown

**Fig. 5:**
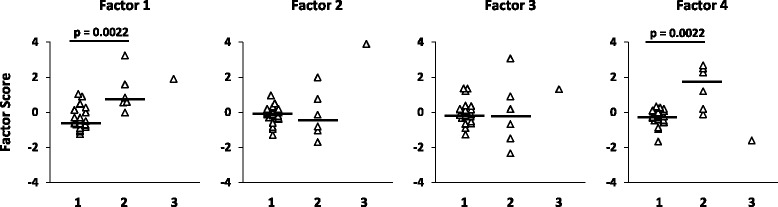
Factor 1 and factor 4 derived from CSF gene expression discriminate patients grouped by cluster analysis. Scores for the four factors defined by factor analysis on CSF gene expression data are shown for each patient classified into clusters 1, 2, or 3 in cluster analysis. Statistically significant differences in factor scores between group 1 (*n* = 24) and group 2 (*n* = 6) patients were assessed by Mann-Whitney test; *p* values ≤0.0125 are shown, *n.s.* not significant. Each *dot* represents the score value for that specific factor in each individual patient; the *line* marks the median value

**Fig. 6:**
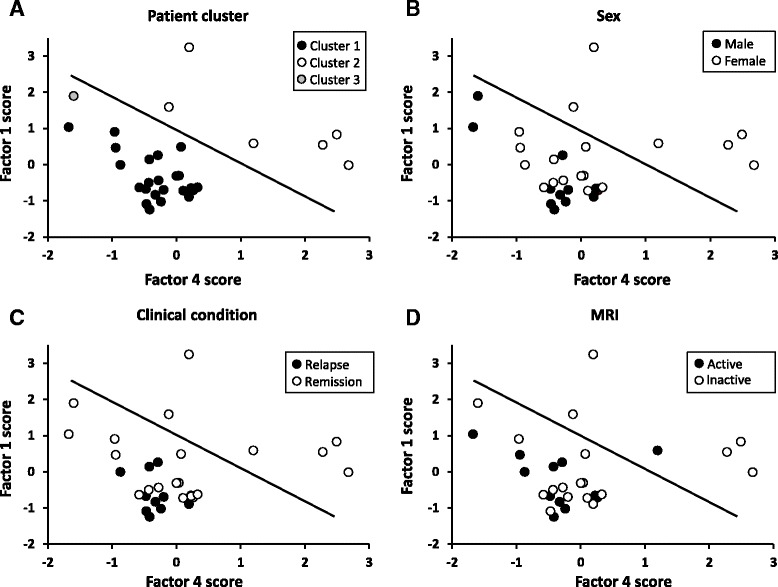
Factor 1 and factor 4 discriminate patients grouped by cluster analysis but not by sex, clinical, or MRI condition. Scatter plots of factor 1 and factor 4 scores in RRMS patients grouped by cluster analysis on CSF gene expression data (**a**), sex (**b**), clinical (**c**), and MRI (**d**) condition are shown. Cluster 1 and cluster 2 patients, but not patients grouped according to the other parameters, distribute in two distinct areas (separated by the *straight line*)

Factor scores were not associated with sex, clinical, or MRI status. However, patient scores for factor 1 and factor 4, but not for factor 2 and factor 3, were significantly higher in cluster 2 than in cluster 1 patients (Fig. 5) with a high discriminating power (AUC >0.90 by ROC curve analysis) (Table 5). These findings suggest enrichment of pathways involved in antiviral/pro-inflammatory (factor 1) and counter-regulatory (factor 4) immune responses in the CSF of cluster 2 patients. A scatter plot visualizing the relationship between factor 1 and factor 4 scores highlights the spatial segregation of cluster 1 and cluster 2 and suggests that assignment of patients to either cluster might depend on the ratio between these two factors (Fig. 6a). Conversely, neither gender nor clinical (relapse/remission) or radiological (presence/absence of gadolinium-enhancing lesions) condition determines such a net separation of patients (Fig. 6b-d). This finding reinforces the idea that the gene signatures discriminating cluster 1 and cluster 2 capture biological processes in CSF that are unrelated to disease characteristics at the time of sampling.

**Table 5:** Discriminatory power for patient clustering of factors derived from CSF gene expression data

Artificial factors	AUC (95 % CI)
Factor 1	0.91 (0.80–1.0)
Factor 2	0.42 (0.06–0.79)
Factor 3	0.45 (0.07–0.83)
Factor 4	0.91 (0.78–1.0)

ROC curve analysis was performed to assess the discriminatory accuracy of factors derived from CSF gene expression data for cluster 1 and cluster 2 patients; cluster 3 patient was not considered

*AUC* area under ROC curve, *CI* confidence interval

**Fig. 7:**
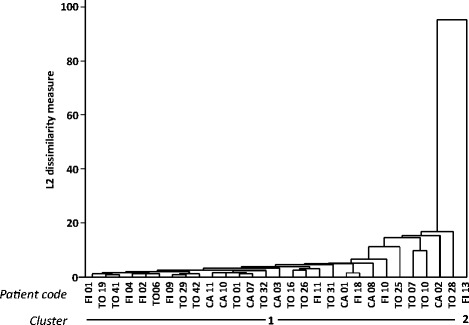
Dendrogram of RRMS patients based on immune gene expression in PBMC. Cluster analysis was carried out on the expression data of 41 immune-related genes obtained in 29 PBMC samples, by using average linkage method with Euclidean similarity measure

By applying cluster analysis to PBMC gene expression data (dendrogram shown in Fig. 7), most MS patients (*n* = 28) clustered into a single group confirming no major differences in gene expression patterns. Only a male patient undergoing a clinical relapse and with an active MRI scan clustered separately. Factor analysis on PBMC gene expression data allowed to identify four factors that altogether explained 63 % of the variability in the dataset (Table 6). Factor 1 was mainly associated with type 1 IFN-related genes (MxA, Usp18, PKR, IFI6, OAS1, IRF7, IFIT1), BAFF, and the T-cell chemoattractant CXCL10, which are also IFN-stimulated genes (factor loadings >0.85), and to a lesser extent, with IL-15, ISG20, iNOS, CD68, and granzyme B. Factor 2 was mainly associated with CD56, NKp46, perforin, IFN-αR1, MHC class II, NAMPT, CD20, and IL-1β (factor loadings >0.70), and to a lesser extent, with CXCL13 and IL-2, suggesting coordinated NK-cell, B-cell, and macrophage activation. ISG20, TNF, CD68, MMP-9, CD8, and to a lesser extent, IL-17A are associated with factor 3, while BDCA-2, FoxP3, and COX-2 (negative factor loading), and to a lesser extent, p40 and IL-2 are associated with factor 4. None of these four factors are associated with demographic, clinical, radiological, or CSF features. However, factor 2 score was markedly higher in the patient clustering separately (score value =5) than in the remaining MS cohort (median score value =−0.23, range −1.0 to +0.24). This finding is discussed more extensively in the next paragraph.

**Table 6:** Factor loadings on PBMC gene expression data

Gene	Factor 1	Factor 2	Factor 3	Factor 4
MxA	0.95			
Usp18	0.94			
PKR	0.94			
IFI6	0.94			
OAS1	0.93			
BAFF	0.91			
CXCL10	0.90			
IRF7	0.90			
IFIT1	0.89			
IL-15	0.61			
CD56		0.97		
Perforin		0.96		
MHC class II		0.96		
NAMPT		0.95		
CD20		0.93		
IL-1β		0.85		
IFN-αR1		0.83		
NKp46		0.74		
CXCL13		0.66		
ISG20			0.75	
TNF			0.75	
CD68			0.69	
MMP-9			0.63	
CD8			0.61	
IL-17A			0.53	
BDCA-2				0.67
FoxP3				0.62
COX-2				−0.62
p40				0.56
IL-2		0.51		0.52
ISG20	0.56			
iNOS	0.55			
CD68	0.54			
Granzyme B	0.53			

Factor loadings >0.5 are shown

### EBV gene expression

To search for a link between the immune-related gene signatures and/or the patient clusters identified by multivariate statistical approaches and EBV infection status, we then evaluated expression of five EBV latent (EBV-encoded small RNA 1 (EBER1), EBV nuclear antigen (EBNA)1, EBNA3A, latent membrane protein (LMP)1, LMP2A) and two EBV lytic (BZLF1, gp350/220) genes in all the analyzable CSF cell and PBMC samples. EBV transcripts were detected in only a few samples: 3 of 31 CSF cell samples (9.7 %) and 4 of 29 (13.8 %) PBMC samples from 5 of 31 patients (16.1 %) (Table 7). All five EBV+ patients belonged to cluster 1, as defined by cluster analysis on CSF gene expression data; of these, three were clinically relapsing/MRI active and two were clinically remitting/MRI inactive (Table 7). Relative to GAPDH, the frequency of viral transcripts was hundred- to thousand-fold lower than that of CD19 and CD20 (pre-amplified in the same cDNA aliquot). It is worth noting that GAPDH Ct values in the three EBV+ CSF samples ranged between 17.3 and 18.2, while the median GAPDH Ct value in the MS cohort was 20.0 (range 14.2–27.7), with values >18.5 being detected in 71 % (22 of 31) of CSF samples (Table 7). This suggests that, despite enrichment of target genes by pre-amplification, low frequency viral transcripts could be missed in most CSF samples because of insufficient material.

**Table 7:** EBV gene expression levels and infection programs detected in CSF cells and PBMC from five RRMS patients

Patient code/sex, age	Time since diagnosis (months)	EDSS	Clinical condition	Gadolinium enhancement on MRI	IgG index	Cells/μl CSF	CSF					PBMC				
							EBV RNA speciesa	EBV infection program	CD19 RNA^a^	CD20 RNA^a^	GAPDH RNA (Ct)	EBV RNA species^a^	EBV infection program	CD19 RNA^a^	CD20 RNA^a^	GAPDH RNA (Ct)
FI01/F 25 years	<1	1	Relapse	Present	0.90	2	n.d.	–	0.02	0.02	20.5	EBER1 0.00003	Latency 0 program	0.05	0.19	13.7
TO41/F 37 years	15	1	Remission	Absent	2.07	14	EBER1 0.00015	Latency II or default program	0.08	0.18	17.4	EBER1 0.000017	Latency 0 program	0.008	0.35	13.6
							LMP2A 0.0001									
TO32/F 35 years	5	2	Relapse	Present	0.67	10	LMP1 0.00023	Latency II or default program	0.05	0.13	18.2	n.d.	–	0.08	0.20	15.6
CA07/F 22 years	17	1	Remission	Absent	0.78	5	n.d.	–	0.13	0.19	19.7	LMP1 0.00008	Latency II or default program	0.04	0.37	16.8
FI13/M 51 years	<1	1	Relapse	Present	0.81	7	EBER1 0.0003	Lytic program	0.01	0.07	17.3	EBER1 0.00035	Latency III or growth program	0.07	11.7	18.6
							BZLF1 0.00009	(immediate early gene)				EBNA1 0.0004				
												EBNA3A 0.0004				
												gp350/220 0.0012				

Demographic, clinical, MRI, and CSF data of five RRMS patients with detectable EBV transcripts in CSF cells and/or PBMC are shown together with expression values of individual viral genes, B-cell related genes (CD19, CD20), and the housekeeping gene GAPDH in the corresponding samples

*n.d.* not detected

^a^Gene expression values are presented as 2^-ΔCt relative to GAPDH

The pattern of EBV gene expression observed in five MS patients was highly variable (Table 7). In PBMC from two female patients, one relapsing/MRI active (FI01) and one remitting/MRI inactive (TO41), detection of the untranslated EBV transcript EBER1, in the absence of any other viral latent gene product, suggests latency 0 program. This EBV gene program is expressed in infected circulating memory B cells and allows the virus to escape immune surveillance [43]. Of these two patients, only TO41 also displayed EBER1 in CSF cells together with the EBV latent gene LMP2A. Expression of LMP2A in the absence of EBV nuclear antigens suggests latency II or default program [43]. EBV latency activation (latency II program) manifesting as expression of LMP1, another gene encoding a latently expressed membrane protein, was found also in CSF cells of a relapsing/MRI active female patient (TO32) and in PBMC of a remitting/MRI inactive female patient (CA07) (Table 7). LMP1 and LMP2A play an important role in supporting the survival and differentiation of EBV latently infected B cells by mimicking CD40 and B cell receptor stimulation, respectively [56–58]. Only a relapsing/MRI active male patient (FI13) displayed detectable levels of EBV genes that are expressed during viral reactivation (Table 7). BZLF1, which is associated with the immediate early lytic cycle and plays a key role in the switch from latent to lytic infection, was detected in CSF cells while gp350/220, which is associated with the late lytic cycle and encodes a major EBV envelope glycoprotein [43, 59], was detected in PBMC suggesting virion production (Table 7). In this patient, profound deregulation of EBV infection in PBMC was also revealed by the presence of EBNA-1 and EBNA-3A RNA (latency III or growth program) [43] and strikingly elevated levels of CD20 RNA (25-fold higher that the median value in the cohort), suggesting new infection events and B-cell activation, respectively (Table 7). It is worth noting that the only patient showing EBV reactivation was the same clustering separately from the rest of the cohort and displaying the highest factor 2 score based on immune-related gene expression data in PBMC (see Fig. 7). This finding provides an explanation for the strong association of factor 2 with genes encoding NK cell-related and cytotoxicity markers (CD56, NKp46, perforin, IFN-αR1), pro-inflammatory molecules (NAMPT, MHC class II, IL-1β, CXCL13) and, as mentioned above, the B-cell marker CD20 as an indicator of EBV-driven B-cell expansion/activation.

## Discussion

The establishment of a relatively simple procedure to perform large-scale gene expression studies in both CSF and peripheral blood is an important step forward towards a better understanding of immunopathological mechanisms and biomarker identification in MS. Here, we have explored the reliability and usefulness of PreAmp real-time RT-PCR to analyze expression of 41 immune-related genes and 7 EBV genes expressed during viral latent and lytic infection in CSF cells and PBMC obtained from 31 therapy-free RRMS patients with relatively short disease duration since diagnosis (median time =12 months). Due to improved sensitivity, PreAmp RT-PCR allowed relative quantification of low level cellular transcripts, such as IL-2, IL-4, IL-6, p40, IL-15, and IL-17A, which are usually undetectable in CSF and/or peripheral blood cells from MS patients using conventional RT-PCR methods. By confirming well-established differences in immune cell composition and mediators of immune responses between CSF cells and PBMC and the association of B-cell/plasmablast enrichment with inflammatory CSF parameters, like CSF cell counts and IgG index, this preliminary study indicates that Pre-Amp RT-PCR can provide reliable information on the abundance and activation status of different immune cell types in both the innate and adaptive branch.

Univariate analysis revealed no or only minor differences in immune-related gene expression between MS patients stratified by sex, clinical, or MRI status. Higher expression of CD4 in CSF cells from female and remitting patients suggests a relatively higher frequency of T helper cells, the predominant population in the CSF, compared to male and relapsing patients. In PBMC, higher expression of BDCA-2 during clinical remission and of IL-10 during clinical relapse may reflect an increased frequency of circulating plasmacytoid DC, the main source of type-1 IFN, and activation of immune regulatory/suppressive mechanisms, respectively. To date, no relevant differences in gene expression have been reproducibly demonstrated in whole blood cells or PBMC when comparing MS patients and controls, patients in clinical relapse and remission, or patients with different disease courses [15–21]. A higher type-1 IFN signature has been detected in the blood, specifically in monocytes [60, 61], of a subset of treatment naïve patients with RRMS and has been associated with a poor response to IFN-β [60, 62, 63]. Recently, differences in PBMC gene expression profiles were detected between male and female patients with RRMS [44], although results interpretation is complicated by different treatment regimens.

Owing to the multivariate dimension, cluster and factor analyses of immune-related gene expression data yielded more relevant results, allowing for gene signature-based subgrouping of patients and interpretation of underlying biological processes and their interaction. The results obtained with the multivariate approach completely differed between CSF cells and PBMC, confirming poor correlation between intrathecal and systemic immune responses. Cluster analysis carried out on CSF gene expression data yielded three clusters of patients. Of these, cluster 1 and cluster 2 (representing 77 and 19 % of the study population, respectively) significantly differed by gene expression but not by sex, clinical condition, disease activity on MRI, or inflammatory CSF parameters. Specifically, cluster 2 showed relatively higher expression of genes encoding MHC class II, macrophage (CD68) and T helper cell (CD4) markers, the type 1 IFN-regulated molecule OAS1, indicators of inflammation (COX-2, NAMPT), and the macrophage-derived pro-inflammatory cytokine IL-1β. By factor analysis, correlated genes were grouped into artificial factors providing information on specific biological processes. Of these, two factors potentially mirror different and interacting biological processes that predominate in the CSF of cluster 2 compared to cluster 1. Factor 1 strongly associates with genes related to a type-1 IFN response (IRF7, MxA, PKR, Usp18, OAS1, IFI6, IFIT1, IFN-αR1, BAFF, BDCA-2), cytotoxic/Th1 T-cell activation (CD8, IFN-γ), and inflammation (NAMPT, COX-2), while the main contribution of CD68, IL-10, MHC class II, and CD4 to factor 4 likely reflects an inhibitory circuit involving immune regulatory cells. This interpretation, along with the finding that assignment of patients to cluster 1 or cluster 2 depends on the ratio between factor 1 and factor 4, is consistent with a tight balancing of pro- and anti-inflammatory immune responses in CSF. Because ROC analysis showed excellent accuracy of some differentially expressed genes, particularly MHC class II, CD4, CD68, and OAS1, as well as of factor 1 and factor 4, in classifying cluster 1 and cluster 2 patients, future studies should ask whether these CSF gene signatures, alone or in combination, may have a prognostic value or be useful to predict a therapeutic response. Multivariate analysis carried out on PBMC gene expression data neither allowed patient clustering nor revealed any significant association of immune-related genes grouped into artificial factors with demographic, clinical, MRI, or CSF characteristics.

Despite the use of an enhanced RT-PCR method, EBV RNA was detected in a minority of CSF cell (10 %) and PBMC (14 %) samples obtained from RRMS patients, a finding that is in line with most previous studies assessing EBV DNA load in MS [37–40]. As we have shown that EBV gene expression was detectable only in CSF cell samples with higher RNA content (GAPDH Ct values <19), it cannot be excluded that low RNA amount remains a major limiting factor for accurate evaluation of EBV infection status in CSF. However, it is worth noting that in all CSF samples (*n* = 3) and half of the PBMC samples (two out of four) with detectable viral RNA, EBV gene expression was indicative of a deregulated infection. Perturbation of EBV infection was inferred by detection of gene products that are associated with different phases of viral latency activation and lytic cycle and are usually not detected in healthy subjects, even when using highly sensitive PCR techniques [64, 65].

Higher antibody- and T-cell-mediated immune responses to EBV in MS patients than in control subjects indicate that EBV infection is perturbed in MS [27–29]. Low prevalence of EBV nucleic acids in CSF and peripheral blood of MS patients and absence of marked differences in EBV DNA load between MS patients and controls [37–40] indicate no persistent or substantial EBV perturbation in these body fluids. However, a higher EBV DNA load was found in PBMC of patients with CIS [66] and during MS clinical exacerbations when serial blood samples were analyzed [38, 41, 42]. Furthermore, a significantly higher incidence of EBV-induced B-lymphocyte transformation in MS patients compared to healthy subjects supports the presence of higher numbers of circulating EBV latently infected B cells in MS [67, 68]. In normal conditions, the EBV life cycle mainly occurs inside the lymphoid tissue, particularly in mucosa-associated lymphoid tissue, like tonsils, where EBV reactivation can occur at very low frequency in plasma cells leading to release of viral particles; the released virus can infect naïve B cells and establish latency in memory B cells [43, 69]. Latently infected memory B cells leaving the lymphoid tissue and entering the blood circulation are extremely rare and shut down expression of viral genes and proteins to avoid detection by cytotoxic T cells, thereby maintaining a life-long infection [70]. Asymptomatic EBV reactivation in healthy individuals may lead to an increase in viral DNA load in the blood in the absence of detectable EBV latent and lytic transcripts, reflecting viral replication in remote lymphoid tissue [64]. The presence of EBV latent and lytic transcripts in CSF cells and/or PBMC from a minority of MS patients described in this study should be interpreted as perturbance of the normal EBV life cycle, which may be transient and therefore difficult to capture in cells circulating through these body fluids, particularly in studies with a single sampling design. The results obtained in autoptic tissue samples, though still controversial [30–32], suggest that an active EBV infection in MS could be mainly confined to brain intraparenchymal perivascular spaces, subarachnoid space where B cells accumulate and organize into B-follicle-like structures [30], and/or CNS-draining lymph nodes [71]. Selective enrichment of CD4+ and/or CD8+ T cells specific for EBV antigens in the CSF of patients with CIS and definite MS has been demonstrated in several studies, supporting the idea of a localized T-cell response to EBV in MS [33, 35, 36].

Due to the low prevalence of EBV RNA+ samples in the analyzed MS cohort, the putative link between EBV infection status, cellular gene expression, and MS disease features could not be evaluated. However, it is worth noting that the only patient displaying EBV reactivation in CSF cells and PBMC was clinically and radiologically active. In PBMC from this patient, a transcript profile suggestive of profound deregulation of viral latency (EBNA-1/EBNA-3A) and virion production (EBER/gp350/220 RNA) was accompanied by the activation of a cellular transcript profile (CD20, CD56, NKp46, perforin, IFN-αR1, MHC class II, NAMPT, IL-1β, CXCL13) that is compatible with B-cell expansion and early induction of a robust innate immune response by EBV reactivation [72]. Productive EBV infection in the peripheral blood in the presence of a disrupted blood-brain barrier could facilitate entry of viral particles and/or newly infected B cells into the CNS. It is intriguing that in CSF cells from the same patient, only BZLF1 RNA, which is associated with the EBV early lytic cycle, was detectable in the absence of any comparable sign of immune arousal. This finding may suggest abortive EBV reactivation and impaired/delayed virus recognition by the immune system in CSF compared to the peripheral blood. A longitudinal study with serial PBMC sampling could help understand whether EBV reactivation recurs in the peripheral blood of MS patients and is associated with immune-related gene signatures and disease activity. It is envisaged that MS patients displaying more frequent EBV reactivation could benefit more from the treatment with last generation B-cell depleting antibodies [5] or antiviral drugs [73, 74].

## Conclusions

This study has allowed to identify PreAmp RT-PCR as a reliable method to carry out large-scale gene expression analyses in paired CSF cell and PBMC samples from MS patients. The results obtained should be interpreted with caution due to the small number of patients included in this study. Investigation of a larger number of immune-related and viral genes in independent patient cohorts is warranted to explore further the usefulness of this method to pinpoint dysimmune processes and alterations in the EBV-host immune system balance in MS. A prospective study will help understand whether CSF gene signatures picked up at diagnosis/early disease stages could be of prognostic value and aid early treatment decisions.

## Additional files

Additional file 1:
**List of Taqman inventoried assays used to study cellular gene expression.** The table lists the immune-related cellular genes and the corresponding Taqman inventoried gene expression assays used in this study. Additional file 2:
**List of Taqman self designed primers and probes used to study EBV gene expression.** The table lists the EBV genes, the GenBank nucleotide sequence accession numbers, and the self designed primers and probes used in this study to analyze EBV gene expression.

## Abbreviations

AUC: area under ROC curve
BAFF: B-cell activating factor
BCMA: B-cell maturation antigen
BDCA-2: blood dendritic cell antigen 2
CI: confidence interval
CNS: central nervous system
COX-2: cyclooxygenase-2
CSF: cerebrospinal fluid
Ct: threshold cycle
CXCL10: C-X-C motif chemokine 10
CXCL13: C-X-C motif chemokine 13
EBER1: EBV-encoded small RNA 1
EBNA: EBV nuclear antigen
EBV: Epstein-Barr virus
FoxP3: forkhead box P3
gd: gadolinium
IFI6: IFN-α-inducible protein 6
IFIT1: IFN-induced protein with tetratricopeptide repeats
IFN-αR1: IFN-α receptor 1
IFN-γ: interferon-γ
IL: interleukin
iNOS: inducible nitric oxide synthase
IRF7: interferon regulatory factor 7
ISG20: interferon-stimulated exonuclease gene 20 kDa
LCL: lymphoblastoid cell line
LMP: EBV latent membrane protein
MHC class II: major histocompatibility complex class II
MMP-9: metalloprotease-9
MRI: magnetic resonance imaging
MS: multiple sclerosis
MxA: myxovirus (influenza virus) resistance protein
NAMPT: nicotinamide phosphoribosyltransferase
NK cell: natural killer cell
NKp46: NK cell p46-related protein
OAS1: 2′-5′-oligoadenylate synthetase 1
PBMC: peripheral blood mononuclear cell
pDC: plasmacytoid dendritic cell
PKR: protein kinase R
PreAmp: pre-amplification
RRMS: relapsing-remitting MS
RT-PCR: reverse-transcription polymerase chain reaction
TNF: tumor necrosis factor
TLR: Toll-like receptor
Usp18: ubiquitin-specific peptidase 18
