# Supplementary material for: Immune and Epstein-Barr virus gene expression in cerebrospinal fluid and peripheral blood mononuclear cells from patients with relapsing-remitting multiple sclerosis
Source: J Neuroinflammation. 2015 Jul 14;12:132. doi: 10.1186/s12974-015-0353-1 (PMC4501166; doi:10.1186/s12974-015-0353-1)
Supplement: Additional file 1: — List of Taqman inventoried assays used to study cellular gene expression. The table lists the immune-related cellular genes and the corresponding Taqman inventoried gene expression assays used in this study. [file 12974_2015_353_MOESM1_ESM.docx]

**List of Taqman inventoried assays used to study cellular gene expression**

| Gene | Assay code | Gene | Assay code |
| --- | --- | --- | --- |
| Glyceraldehyde 3-phosphate dehydrogenase (GAPDH) | Hs99999905_m1 | IL-6 | Hs00985639_m1 |
| CD20 | Hs00544818_m1 | IL-10 | Hs00961622_m1 |
| CD19 | Hs00174333_m1 | IL-15 | Hs01003716_m1 |
| CD138 | Hs00896423_m1 | IL-17A | Hs00174383_m1 |
| B cell maturation antigen (BCMA) | Hs03045080_m1 | Interleukin 12B or p40 | Hs01011518_m1 |
| CD4 | Hs01058407_m1 | C-X-C motif ligand 10 (CXCL10) | Hs00171042_m1 |
| CD8 | Hs00233520_m1 | CXCL13 | Hs00757930_m1 |
| CD56 | Hs00941830_m1 | Interferon regulatory factor 7 (IRF7) | Hs00185375_m1 |
| Natural Killer Cell p46-related protein (NKp46) | Hs00183118_m1 | Interferon Stimulated Exonuclease Gene 20kDa (ISG20) | Hs00158122_m1 |
| CD68 | Hs00154355_m1 | IFNα-inducible protein 6 (IFI6) | Hs00242571_m1 |
| Forkhead box P3 (Foxp3) | Hs01085834_m1 | Myxovirus (influenza virus)  resistance protein (MxA or Mx1) | Hs00895608_m1 |
| Blood dendritic cell antigen 2 (BDCA-2) | Hs01092462_m1 | Protein kinase R (PKR) | Hs00169345_m1 |
| Perforin | Hs00169473_m1 | 2'-5'-oligoadenylate synthetase 1 (OAS1) | Hs00973637_m1 |
| Granzyme B | Hs01554355_m1 | Interferon-induced protein with  tetratricopeptide repeats 1 (IFIT1) | Hs01675197_m1 |
| Matrix metalloproteinase-9 (MMP-9) | Hs00234579_m1 | Ubiquitin specific peptidase 18 (Usp18) | Hs00276441_m1 |
| Interferon- γ (IFN-γ) | Hs00174143_m1 | IFN-α receptor 1 (IFN-αR1) | Hs01066118_m1 |
| Tumor necrosis factor (TNF) | Hs00174128_m1 | HLA-DR or major histocompatibility  complex II (MHCII) | Hs00219575_m1 |
| Interleukin (IL)-1 β | Hs01555410_m1 | Nicotinamide phosphoribosyltransferase (NAMPT) | Hs00237184_m1 |
| IL-2 | Hs00174114_m1 | B cell activating factor (BAFF) | Hs00198106_m1 |
| IL-4 | Hs00174122_m1 | Inducible nitric oxide synthase (iNOS) | Hs01075529_m1 |
| IL-5 | Hs00174200_m1 | Cyclooxygenase-2 (Cox-2) | Hs00153133_m1 |
