# Supplementary material for: Immune and Epstein-Barr virus gene expression in cerebrospinal fluid and peripheral blood mononuclear cells from patients with relapsing-remitting multiple sclerosis
Source: J Neuroinflammation. 2015 Jul 14;12:132. doi: 10.1186/s12974-015-0353-1 (PMC4501166; doi:10.1186/s12974-015-0353-1)
Supplement: Additional file 2: — List of Taqman self designed primers and probes used to study EBV gene expression. The table lists the EBV genes, the GenBank nucleotide sequence accession numbers, and the self designed primers and probes used in this study to analyze EBV gene expression. [file 12974_2015_353_MOESM2_ESM.docx]

**List of Taqman self designed primers and probes used to study EBV gene expression**

| Gene | GenBank  nucleotide sequence accession number | Forward primer | Reverse primer | FAM-labelled probe |
| --- | --- | --- | --- | --- |
| EBV-encoded small RNA 1 (EBER1) | J02077.1 | GTTGCCCTAGTGGTTTCG | CCCCGGGACTTGACC | ACACACCGCCAACGCTCAGT |
| EBV nuclear antigen (EBNA) 1 | M12553.1 | CCCACCATGGAATCATTTGAAGGA | TCGCCGGTAGTCTGTACATTATCT | CCCCGCTCATTGTC |
| EBNA3A^a^ | AJ507799.2 | GCCCCGTGTCCGGTAG | GAGTTGATCCCCTGGAGATACAG | CACAGGCCCCACCTAC |
| EBV latent membrane protein (LMP) 1 | HM366148.1 | GGACAACGACACAGTGATGAACA | CATCGGTAGCTTGTTGAGGGT | CCACCACGATGACTCC |
| LMP2A | M24212 | GAGATGGCGCCGTTTGAC | TGAGGACAAGTACACATGCCAAAA | ATGCCGCCACAAACA |
| BZLF1 | M17547.1 | CTCAACCTGGAGACAATTCTACTGT | TGCTAGCTGTTGTCCTTGGTTAG | CTGCTGCTGCTGTTTG |
| gp350/220 | E01006 | AGAATCTGGGCTGGGACGTT | ACATGGAGCCCGGACAAGT | AGCCCACCACAGATTACGGCGGT |

^a^ Protein Genbank identifier: CAD53419.1
